# Identifying mosquito plant hosts from ingested nectar secondary metabolites

**DOI:** 10.1038/s41598-025-88933-1

**Published:** 2025-02-22

**Authors:** Amanda N. Cooper, Louise Malmgren, Frances M. Hawkes, Iain W. Farrell, Domonbabele F. d. S. Hien, Richard J. Hopkins, Thierry Lefèvre, Philip C. Stevenson

**Affiliations:** 1 Royal Botanic Gardens Kew, Kew Road, Richmond, Surrey, TW9 3AE UK; 2 Natural Resources Institute, University of Greenwich, Chatham Maritime, Kent, ME4 4TB UK; 3 Institut de Recherche en Sciences de La Santé (IRSS), Bobo Dioulasso, Burkina Faso; 4 Laboratoire Mixte International Maladies à Vecteurs en Afrique de l’Ouest (LAMIVECT), Bobo Dioulasso, Burkina Faso; 5 MIVEGEC, Université de Montpellier, IRD, CNRS, Montpellier, France

**Keywords:** Plant-insect interactions, Plant secondary metabolites, *Culex quinquefasciatus*, *Anopheles coluzzii*, *Lantana camara*, *Ricinus communis*, *Cascabela thevetia*, Ecology, Entomology

## Abstract

Establishing how plants contribute food and refuge to insects can be challenging for small species that are difficult to observe in their natural habitat, such as disease vectoring mosquitoes. Currently indirect methods of plant-host identification rely on DNA sequencing of ingested plant material but are often unsuccessful for small insects that feed primarily on plant sugars or have little contact with plant cells. Here we developed an innovative approach to determine species-specific phytophagy by detecting taxon-specific plant secondary metabolites (PSMs) in nectar. Two mosquito species were exposed to three PSMs, each present in the nectar of a known plant host, firstly from dosed sucrose solutions and secondly from flowers. Both experiments yielded high rates of PSM detection in mosquitoes using liquid chromatography-mass spectrometry (LC-MS). PSMs were consistently detected in mosquitoes up to 8 h post-ingestion. In experiments consisting of two or three plant species, multiple PSMs from different host plants could be detected. These positive results demonstrate that PSMs could be useful indicators of insect plant-hosts selection in the wild. With expanded knowledge of nectar-based PSMs across a landscape, improved knowledge of plant-host relationships could be achieved where direct observations in their natural habitat are lacking. Increasing understanding of vector insect ecology will have an important role in tackling vector-borne disease.

**Supplementary Information:**

The online version contains supplementary material available at 10.1038/s41598-025-88933-1.

## Introduction

Plants play a fundamental role in the ecology of flower visiting insects, providing nectar and pollen for food as well as habitat for reproduction and refuge. Most insects have evolved to utilize multiple plant species, as this provides a competitive advantage but for many insects specific floral nectars and pollen may be preferred^1^. Establishing host plant choice by insects requires either direct observation which is time consuming and prone to missed observations or analysis of plant remnants present on or in the insect post-interaction^2^. DNA barcoding of plant material that is either ingested or found externally on insects, such as pollen, has been used widely to identify insect host plants^3–6^. This approach is successful for insect groups that feed directly on plant cells, such as herbivores and pollen-feeders because it requires DNA ingestion. However, this is more challenging for insects that only feed on plant sugars, such as nectar, as they are unlikely to contain plant DNA^1,7^.

For nectivorous insects that are also pests or disease vectors, exploring complementary analytical methods to identify their plant hosts could offer additional insights. While nectar may include little or no plant DNA, it typically contains plant secondary metabolites (PSMs), which may provide the plant with a defence against antagonists and nutrition or antimicrobial resources for nectar feeding animals^8–12^. PSMs are highly diverse across plant taxa, because they have evolved as a result of defensive adaptations of a highly diverse range of herbivores and diseases, leading to the production of specific and unique compounds throughout the plant, including the nectar^11,13–15^. Consequently, PSMs could be used as chemical markers in nectar feeding insects to determine host plant use in botanically complex landscapes.

To assess whether PSM identification could be used to distinguish the plant species an insect had collected nectar from, we used mosquitoes as a model taxon. This is because mosquitoes mainly depend on floral and extra-flora nectar for their carbohydrate requirements, with males feeding exclusively on such sugar meals and females consuming it alongside the animal blood needed for oogenesis^16–19^. While nectar primarily comprises carbohydrates, the PSMs that are also found in nectar may enhance the nutritive value of nectar^20,21^. Information about preferred plants for mosquitoes in human populated landscapes where malaria is a problem could inform land management and help reduce the burden of malaria disease because abundant natural plant sugars in a landscape can influence traits that play key roles in the spread of malaria (i.e. components of vectorial capacity, namely mosquito density, longevity, blood-feeding rate, competence for *Plasmodium*, and the parasite’s extrinsic incubation period)^22–30^. As well as transmitting malaria, mosquitoes vector a range of viruses, (e.g. chikungunya, dengue, Zika, West Nile, and yellow fever), and other parasites (e.g. round worms)^31–33^. Thus, plant host identification for mosquitoes is vital for improved understanding of mosquito ecology and could inform new avenues for vector control that target mosquitoes during plant interactions. PSM detection may offer a valuable alternative for identifying food plants in cases where DNA barcoding presents challenges (e.g., low identification rates)^7,34–37^.

Indirect analysis of host plants for insects such as mosquitoes is additionally useful because they are small and often crepuscular or nocturnal, making them difficult to observe when feeding from plant hosts. While it is widely assumed that mosquitoes are generalist feeders, they have been observed to preferentially feed on specific plant hosts in laboratory experiments^22,34,38–41^. However, less is known about how mosquitoes use plant hosts in their natural habitat^22^. Cold anthrone testing is typically used to confirm sugar ingestion by mosquitoes^42–45^, and DNA sequencing alongside this has been explored as a method to identify plant hosts but with variable success rates^7,34–37^. Other methods included fluorescent dye application to flowers to mark feeding mosquitoes^29,46^. However, this was potentially biased by application to plants that were already known hosts and would not include situational selection of unknown plants.

The aim of this study was to determine if PSMs could be used to identify plant host selection in mosquitoes post-ingestion. To address this, we reviewed previous studies that identified mosquito plant-host selection, and 104 plant hosts were found. The identified plant species were cross referenced with the living collection at the Royal Botanic Gardens, Kew and from ornamental plants in Bobo Dioulassou, Burkina Faso to identify plants from which nectar samples could be collected. From the plant species that were likely to be available, we included plants that were from different plant families and widely occurring throughout the tropics where mosquito-borne diseases are prevalent. Three plant species met these criteria and were accessible for nectar chemical analysis: *Lantana camara* L., *Ricinus communis* L., and *Cascabela thevetia* (L.) Lippold (syn. *Thevetia peruviana*). These plants are widespread throughout the tropics and subtropics, even classified as invasive in some regions^28,47,48^. *L. camara* and *C. thevetia* are common ornamentals in peri-domestic settings^49,50^. *R. communis* is cultivated for oil production and can grow invasively in wetlands^51,52^. Mosquitoes have been observed feeding on *R. communis* and *L. camara* in laboratory feeding experiments^22,28,45,53,54^. *C. thevetia* has likewise been observed as a mosquito plant host^26,38,55^.

Nectar was collected from the three target plant hosts and unique PSMs present in the nectar of each species were identified as ricinine in *R*. *communis* which was detected at an average concentration of 50 ppm, luteolin in *L*. *camara* at a concentration of 10 ppm, and thevefolic acid B in *C*. *thevetia* at a concentration of 400 ppm^56–60^. Targeting these PSMs, we undertook a series of feeding experiments to address the following questions:

1. Can PSMs present in an experimental sugar solution as an artificial nectar be detected in mosquitoes post ingestion?
2. Can PSMs present in floral nectar be detected in mosquitoes after feeding on flowers in vivo? Further, can multiple PSMs be detected in mosquitoes when multiple nectar sources are present?
3. How long can PSMs be detected for in mosquitoes after ingestion?

## Results

### Experiment 1. Plant secondary metabolite detection in mosquitoes

**Fig. 1:**
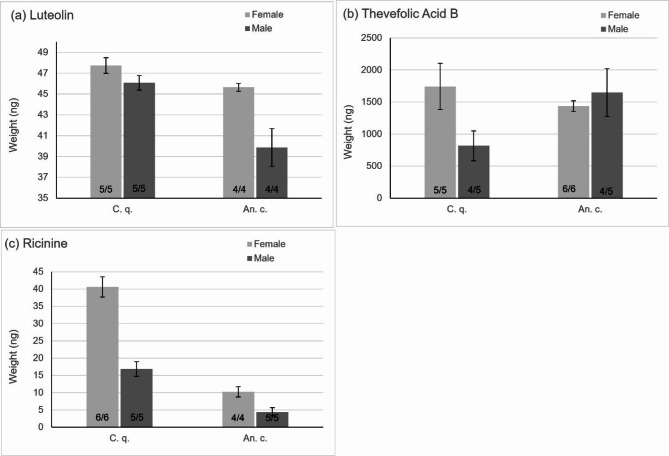
In dosed sugar solution assays, male and female sample mean detected weights of ricinine (**a**), thevefolic acid b (**b**), and luteolin (**c**), in nanograms. Samples of species *Culex quinquefasciatus* (C.q.) and *Anopheles coluzzii* (An. c.) were measured. Error bars show standard error. Number of samples with positive detection out of all samples reported at the base of the column.

All three PSMs were detected in mosquitoes of both sexes of *Cx. quinquefasciatus* and *An. coluzzii* post-ingestion of treatment sugar solutions. Luteolin weights detected ranged from 39.86 to 47.74 ng in samples of five mosquitoes (Fig. 1a). Thevefolic acid B detected weight ranged from 817.02 to 1743.06 ng in samples of five mosquitoes (Fig. 1b). Thevefolic acid B was the only compound that was not detected in one sample of males from both species (Fig. 1b). Ricinine weight detected ranged from 4.44 to 40.60 ng in samples of five mosquitoes (Fig. 1c).

The gradient of concentrations of PSMs provided in dosed solutions, determined by naturally occurring concentrations, was reflected in the variation of weights detected in samples of luteolin and thevefolic acid B. Luteolin, the lowest concentration (35 ng/ul), was detected in low quantities: *Cx*. *quinquefasciatus* (females: 47.75 ± 0.74 ng, males: 46.07 ± 0.69 ng), *An*. *coluzzii* (females: 45.65 ± 0.37 ng, males: 39.86 ± 1.83 ng) (Fig. 1a). Thevefolic acid B, provided at the highest concentration (440 ng/ul), was found at the largest weight in mosquitoes: *Cx*. *quinquefasciatus* (females: 1743.06 ± 360.35 ng, males: 817.02 ± 232.69 ng), *An*. *coluzzii* (females: 1435.65 ± 83.69 ng, males: 1647.98 ± 372.47 ng) (Fig. 1b). Unexpectedly, ricinine, provided at an intermediate concentration (250 ng/ul), had low weight detected in mosquitoes, *Cx*. *quinquefasciatus* (females: 40.60 ± 2.92 ng, males: 16.82 ± 2.09 ng), *An*. *coluzzii* (females: 10.23 ± 1.46 ng, males: 4.44 ± 1.21 ng) (Fig. 1c). No PSMs were detected in any of the male or female control samples from both species.

### Experiment 2. Flowering plant feeding experiment

#### Single plant assay

**Fig. 2:**
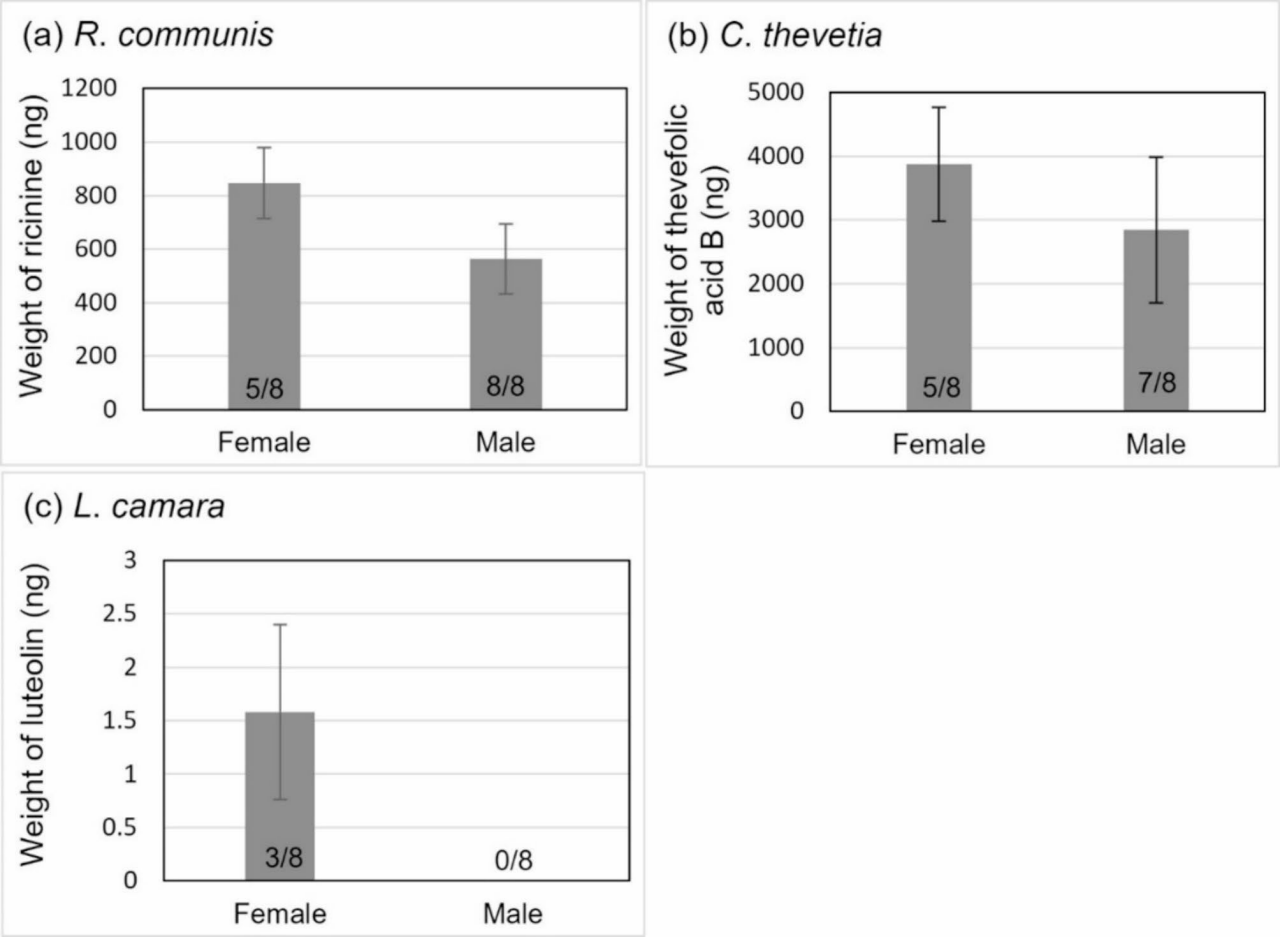
From the single-species flower assays, male and female sample mean detected weight, in nanograms (excluding samples with negative detection): (**a**) ricinine in *R*. *communis*, (**b**) thevefolic acid B in *C*. *thevetia*, and (**c**) luteolin in *L*. *camara*. Error bars show standard error. The number of samples with positive detection out of all samples reported at the base of the column.

Mosquitoes in the single plant assays were found to contain the target PSMs except for luteolin in male mosquitoes. Luteolin was detected in three female samples fed on *L*. *camara* with an average weight of 1.57 ± 1.42 ng (Fig. 2c). Ricinine was detected in all the samples of males and five female samples from insects offered *R*. *communis*. The average weight of ricinine detected in positive samples was 846.30 ± 132.30 ng (females) and 563.24 ± 187.47 ng (males) (Fig. 2a). Thevefolic acid B was detected in five of eight samples of females and seven of eight samples of males that fed on *C*. *thevetia* (Fig. 2b). The average weight of thevefolic acid B detected in positive samples was 3871.79 ± 894.98 ng (females) and 2846.41 ± 1142.72 ng (males).

#### Two-plant assay

**Fig. 3:**
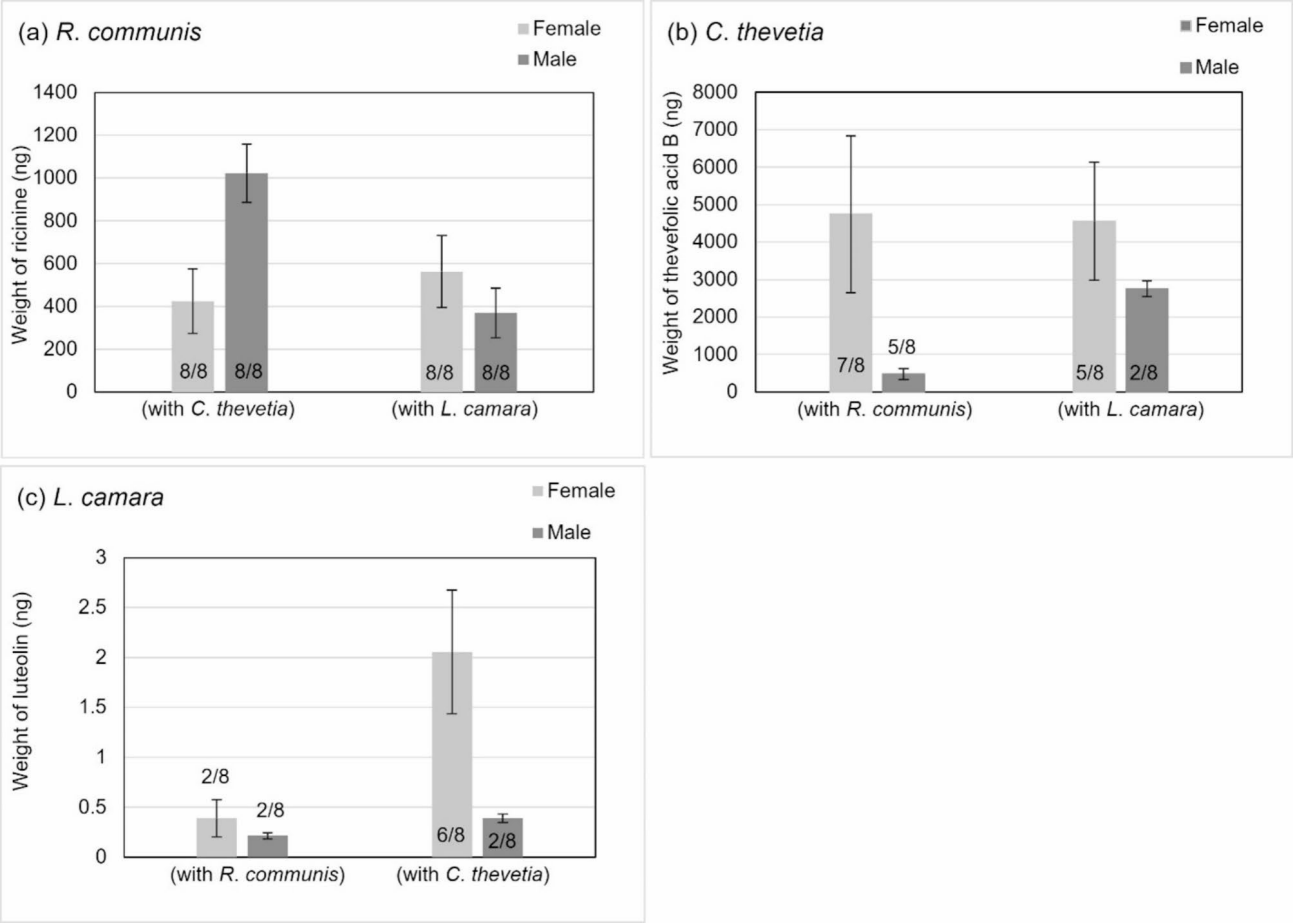
From the two-flower assays, male and female sample mean detected weights (excluding samples with negative detection), in nanograms of: **a**) ricinine in *R*. *communis*, **b**) thevefolic acid B in *C*. *thevetia*, and luteolin in *L*. *camara*. Error bars show standard error. Number of samples with positive detection out of measured samples reported at the base of the column.

In the two-plant feeding assays, all the PSMs were detected in some of the samples. In the feeding assay of *R*. *communis* and *C. thevetia*, both ricinine and thevefolic acid B were detected, with ricinine in all samples (Fig. 3a) and thevefolic acid B detected in seven of eight female samples and four of eight male samples (Fig. 3b). Mean weights of ricinine observed were 424.32 ± 150.63 ng (females) and 1022.92 ± 135.62 ng (males) (Fig. 3a). A higher weight of thevefolic acid B was detected in female samples (4743.96 ± 2095.27 ng) than male samples (475.56 ± 142.93 ng) (Fig. 3b).

In the feeding assay consisting of *C*. *thevetia* and *L*. camara, thevefolic acid B was detected in five of eight female samples and two of eight male samples (Fig. 3b). Luteolin was detected in six of eight female samples and two of eight male samples (Fig. 3c). Mean weight of thevefolic acid B in females was 4560.71 ± 1574.37 ng and in males was 2752.63 ± 212.08 ng (Fig. 3b). Mean weight of luteolin in females was 2.06 ± 0.62 ng and in males 0.30 ± 0.04 ng (Fig. 3c).

In feeding assays of *R*. *communis* and *L*. *camara*, ricinine was detected in all samples whilst luteolin was present in only two of eight samples of both sexes. The mean weight of luteolin was comparable in both sexes, 0.39 + 0.19 ng in females, and 0.21 ± 0.03 ng in males (Fig. 3c). Mean weights of ricinine were also comparable in both sexes, 563.33 ± 168.54 ng (females) and 369.08 ± 116.24 ng (males) (Fig. 3a).

#### Three-plant assay

**Fig. 4:**
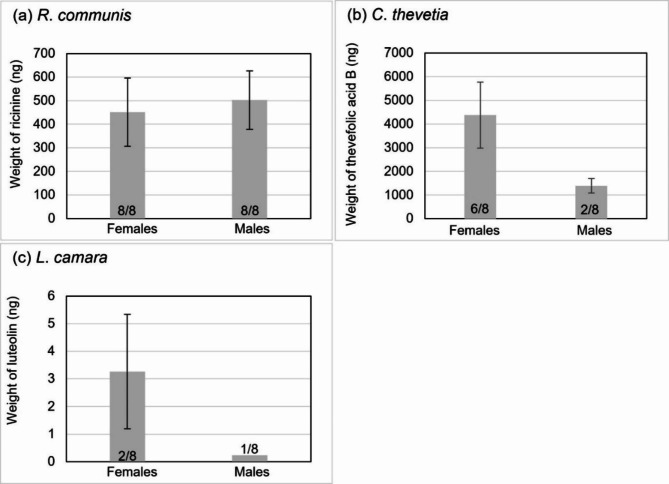
From the three-flower assay, mean detected weight (excluding samples with negative detection) in nanograms of each PSM in males and females: **a**) ricinine in *R*. *communis*, **b**) thevefolic acid B in *C*. *thevetia*, and luteolin in *L*. *camara*. Error bars show standard error. Number of samples with positive detection out of measured samples reported at the base of the column.

Ricinine was detected in all mosquito samples in the three-plant feeding assay, with mean weights of 451.75 ± 144.53 ng (females) and 502.36 ± 123.31 ng (males) (Fig. 4a). Thevefolic acid B was detected in six female samples and two male samples, with mean weights of 4376.96 ± 1391.5 ng (females) and 1392.7 ± 301.45 ng (males) (Fig. 4b). Luteolin was present in the fewest samples, in two female and only one male sample (Fig. 4c). Only a single male sample contained luteolin weighing 0.23 ng and mean weight for the two female samples was 3.27 ± 2.07 ng (Fig. 4c). The ranges of weights in all samples of each PSM was comparable to values observed in single and two-plant feeding assays.

### Experiment 3. PSM residence time

**Fig. 5:**
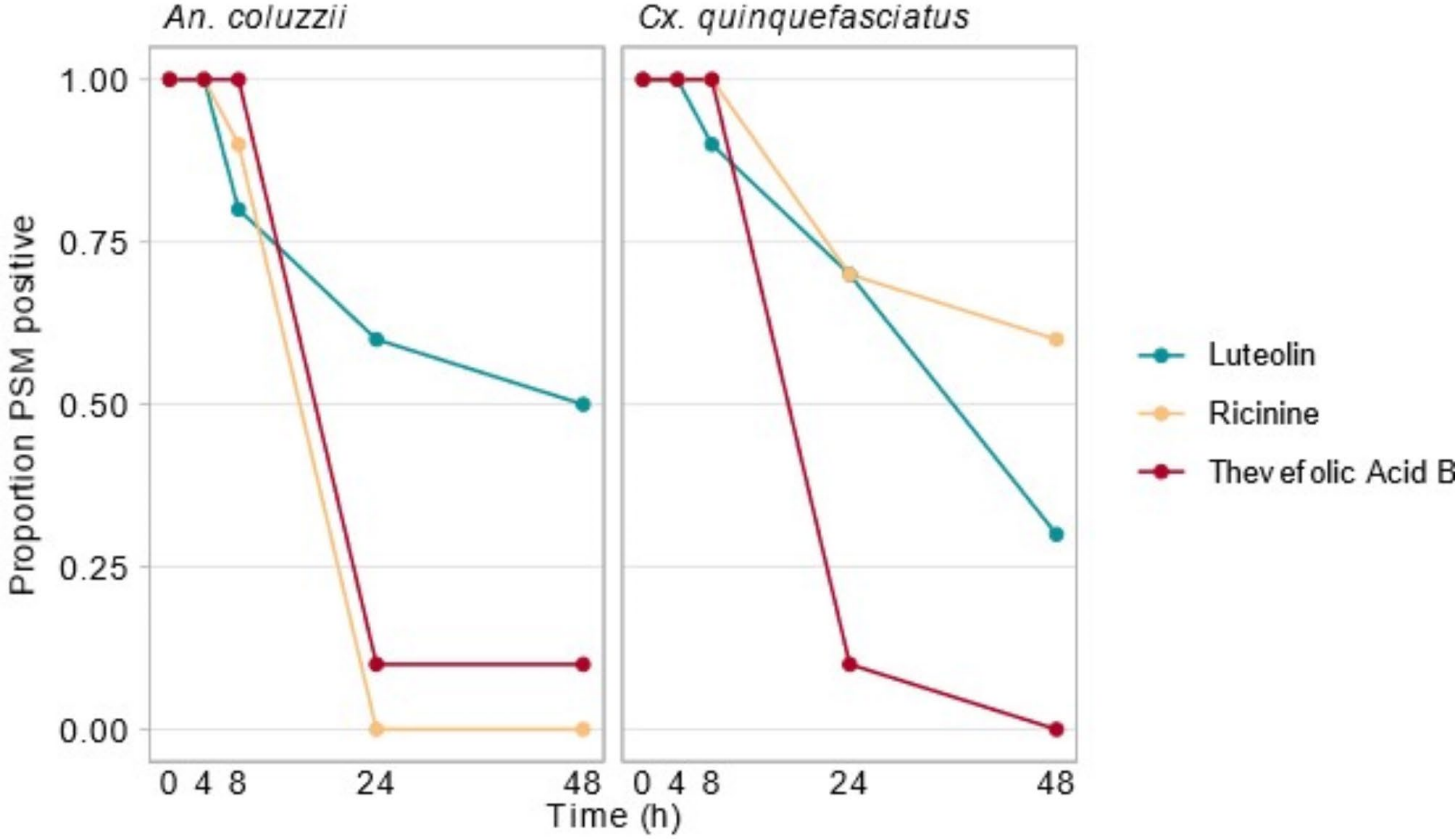
The proportion of individuals of* An*. *coluzzii* and *Cx*. *quinquefasciatus* that were PSM positive at each time point, following the ingestion of a replete sucrose meal. Sexes have been pooled.

**Fig. 6:**
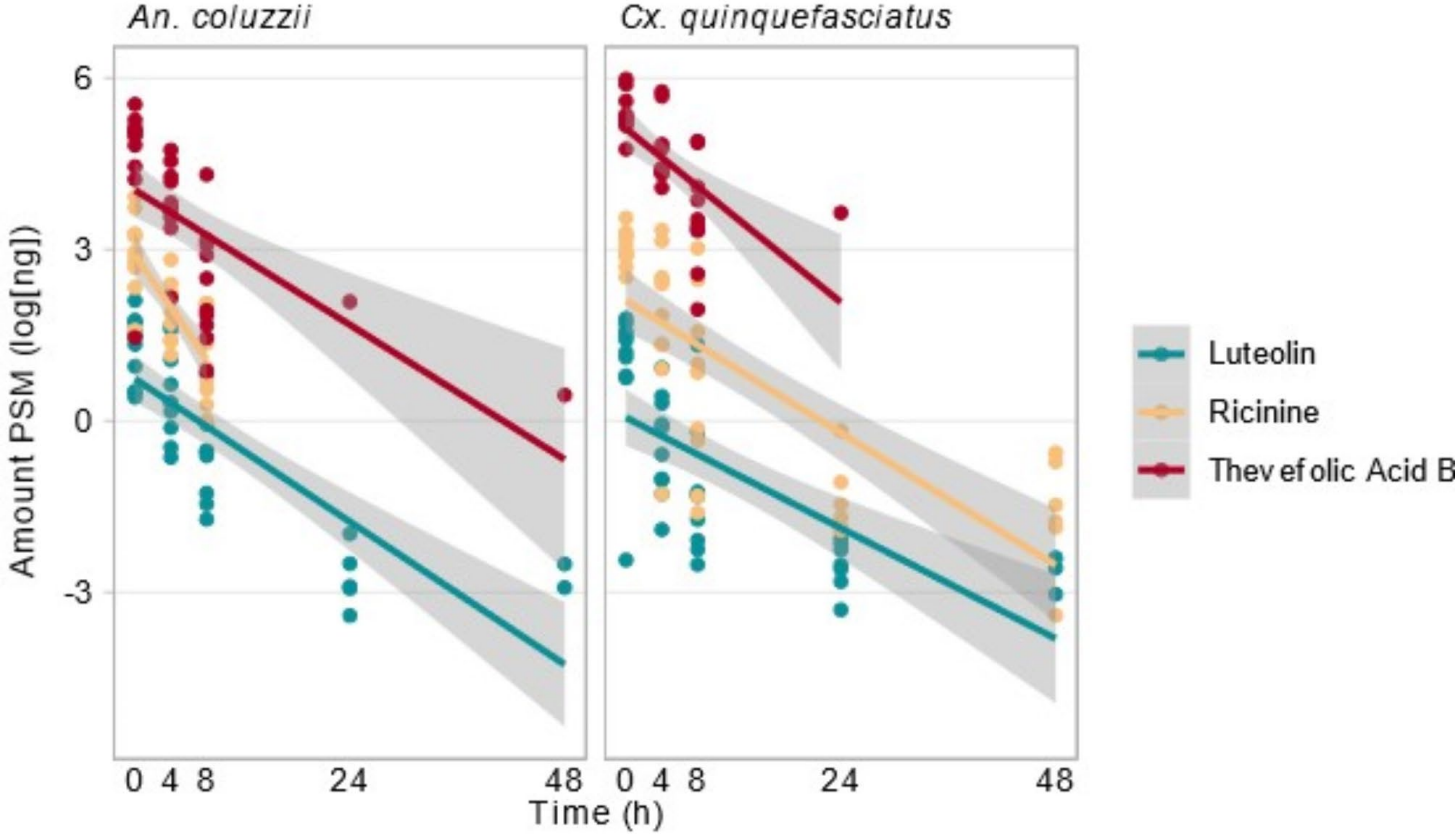
The amount of PSMs (log(ng)) detected in *An*. *coluzzii* and *Cx*. *quinquefasciatus* over time, following their ingestion with a replete sucrose meal. Sexes have been pooled.

The PSMs luteolin, ricinine, and thevefolic acid B were detected in all individuals of *An. coluzzii* and *Cx*. *quinquefasciatus* up to 4 h after ingestion of a replete treated sucrose meal. Most individuals had detectable levels of PSM 8 h after ingestion, after which positive detection declined (Fig. 5). Notably, luteolin, provided at the lowest concentration, was detected in some individuals of both sexes of *An*. *coluzzii* and *Cx*. *quinquefasciatus* throughout the assayed time (Fig. 6). Conversely, thevefolic acid B was only present intermittently in a limited number of individuals 24 and 48 h after feeding. Ricinine was absent in all individuals of *An*. *coluzzii* collected 24 h post-ingestion and onwards but was present in some individuals of *Cx*. *quinquefasciatus* for the duration of the assay.

As in the preceding experiments, the concentration of each PSM present in the sugar treatments were reflected in the amounts detected in the mosquito samples, with the smallest quantities recorded of luteolin (3.51 ± 0.85 ng, female *An*. *coluzzii*) and the largest of thevefolic acid B (280 ± 43.1 ng, female *Cx*. *quinquefasciatus*) (Fig. 6; Table S3). Ricinine, offered at an intermediate concentration (40 mg l-1), was likewise detected at intermediate quantities (the greatest at 28.6 ± 7.63 ng in female *An*. *coluzzii*).

## Discussion

This study demonstrated that PSMs could be detected in mosquitoes post-ingestion, illustrating that PSMs can be used as indicators of plant host selection by insects. All PSMs tested were observed in males and females of both species when fed on dosed sugar solutions and was further validated through PSM detection from in-vivo flower assays. Additionally, this study demonstrated consistent PSM detection at naturally occurring concentrations over an 8-hour period. The detection of multiple PSMs in insects that were offered flowers from two or three plant species suggests that a holistic view of plant host selection could be inferred from PSMs. In flower feeding assays, ricinine was the most frequently detected PSM, which is consistent with behavioural evidence that mosquitoes preferentially feed on *R*. *communis* over *L*. *camara*^22,28,45^. While *C*. *thevetia* has not been compared to *R. communis* or *L*. *camara*, this study demonstrated that it is utilized in the presence of the other plant species. The flower feeding assays suggest a difference in PSM detection between males and females, indicating that plant host selection may differ between the sexes depending on the plant species composition within a landscape. This should be explored further in behavioural choice experiments along with PSM analysis to demonstrate that this method could be used to indicate mosquito feeding preferences for specific plants. For insects that do not consume plant DNA, this method of PSM detection could be an effective, efficient, and high-resolution method for identifying plant hosts in the wild.

Plant host identification methods have previously been explored through the detection of carbohydrates and plant DNA. Carbohydrates in mosquitoes indicate general phytophagy, but lack of sufficient variation in abundance and prevalence in nectar make these compounds unreliable indicators of plant host species^61–63^. Whereas chromatography (GC) was applied to successfully differentiate plant hosts based on constituent sugars of nectar from several plant species it was found 44% of the identified GC profiles matched more than one plant species^22^. Unidentified compounds were also detected that did not correspond to sugar standards and were speculated to be PSMs. DNA barcoding explored in mosquitoes has also had low host plant identification rates, ranging from a success rate of 1.9–41% of plant DNA amplification^7,34,35,37^ and the proportion of simultaneous fructose-positive mosquitoes differed from DNA-positive mosquitoes. Plant DNA likely occurs in mosquitoes because of specific feeding behaviours whereby mosquitoes directly pierce plant cellular tissue that contains DNA, which is exhibited when floral nectar resources are scarce in the landscape^7^. When fed on sugar feeders containing the PSMs, ricinine and luteolin were detected in all mosquito samples (100%) of both sexes and species. Thevefolic acid B was detected in all female mosquito samples (100%) of both species and four of the five (80%) male samples from both species. These are higher detection rates than reported for plant DNA. When flower material was provided for feeding, there was more variability in positive detection, which could be explained through differences in feeding some of the samples and were absent from the control mosquitoes. Moreover, PSMs remained detectable up to 8 h post-feed in experiment 3. Thus, PSM detection could be a more consistent approach to identifying nectar plant hosts than current methods.

While these analyses demonstrated effectively that nectar PSMs can be detected in insects post ingestion further exploration is needed to develop this approach for a range of insect plant host interactions and a wider range of compounds. Luteolin for example is found in several plant taxa so may be less diagnostic is used alone, while ricinine has a more restricted distribution in *R. communis* and related species, including *Tetracarpidium conophorum*^64^, but thevefolic acid B has only been detected in nectar of *C*. *peruviana*^56,65^, indicating it is highly specific. For this study, a single compound was selected as the few studies that have characterized the PSMs in nectar across differing plant taxa found most compound were observed in a single species^11,66^. Further evaluation of nectar chemistry from all plants within a landscape is needed to distinguish the sugar resources available within mosquito foraging habitat^20,66–68^. In the instances where PSMs are found in multiple plants, detection of several PSMs and PSM finger prints or chemical profile within nectar could enable more specific discrimination of plant hosts, as has previously been achieved for sugars^15,22^. One potential challenge is the presence of microorganisms in nectar, which have associations with specific plant communities^12,69,70^ and may interact with and alter the nectar chemistry and should be considered in the implementation of this method^71^. The role of olfactory detection and its relationship to plant host preference should be evaluated as it may correlate with the beneficial properties of plant sugar selection^19,34,41,72–74^.

Plant host identification through PSM detection can provide several avenues for improved understanding of insect ecology. This approach would enable further exploration of plant host selection in nature, accounting for variation in local plant composition and diversity^17,29^. The use or avoidance of particular plants is relevant to insect health and fitness overall because phytophagy not only provides carbohydrates but may be driven by the need for other nutrients^20,75–77^. PSMs present in nectar serve differing functions, such as inhibiting microbes that might degrade nectar quality^14^, and their consumption by insects may even provide a means of self-medication^10,12,78–80^. Alternatively, PSMs may be produced for plant defence and thus may negatively impact insects when PSMs are toxic^14,81–83^. For example, *R*. *communis* is known as a mosquito plant host that can increase survival of *An*. *gambiae* s.s^21,22,34,45,53,84–87^. However, ricinine, can be toxic to mosquitoes having been reported, to increase mortality in *An*. *gambiae* s.s. when continually ingested^28,59,88^. This study observed a rapid decline of ricinine in assayed mosquitoes, with trace amounts detected after 48 h in *Cx. quinquefasciatus* and 8 h in *An. coluzzii*. This decline has been attributed to mosquitoes ability to break down ricinine^59,89^. Further, in malaria vectoring mosquitoes, ricinine consumption significantly shortened the extrinsic incubation period of *Plasmodium* in the mosquito midgut^90^, potentially increasing vector competence where *R*. *communis* is abundant in the landscape. Knowledge of PSM presence and factors that contribute to PSM concentration variability in nectar across all flowering plants would provide greater insights of the role of PSMs in insect life cycles and the transmission of the pathogens they vector^14^.

Within the medical entomology context, *An*. *coluzzii* and *Cx*. *quinquefasciatus*, are vectors of diseases that are increasingly difficult to control; mainly through the use of indoor residual spraying and insecticide-impregnated bed nets that aim to disrupt blood-feeding^33,91^. Knowledge of plant hosts could highlight controls that target mosquitoes during phytophagy and resting^92–95^. For example, vector female mosquito density was reduced by 69.4% where known plant hosts were removed from several villages in Africa^96^. Attractive targeted sugar bait (ATSB) application relies on a good knowledge of plant hosts throughout the landscape^29,97–99^ and has effectively eliminated local mosquito populations in desert habitats where sugar sources are relatively few^100^. Sissoko et al., (2019) also observed a 70-fold and 10-fold decline of female *Aedes aegypti* in sugar rich and sugar poor environments, respectively, when ATSB was applied to known plant hosts^29^.

Accurate identification of plant hosts and their distribution in a landscape may enable better insect species distribution modelling, particularly disease vectors in epidemiological models. Vector distribution models are mainly based on climate and environmental variables, particularly at regional scales, with vegetation generalized by coarse satellite data^101–105^ or land cover classification that do not represent plant species composition^106,107^. Predictive mosquito distribution models are used to identify areas where disease spread and have improved as increasingly fine scales of environmental and ecological data has become available^95^. Incorporating distributions of identified plant hosts are needed to improve current models^92,95,108^. These could extend further to account for sugar availability and nutritional quality in a landscape^24,109^. Also, the impact of PSM-based plant resources will likely predict variation in infection prevalence and intensity, parasite development and mosquito survival and fecundity, which could improve epidemiological models^26^.

## Conclusion

The detection of PSMs ingested in plant sugars offers a promising and novel approach to the study of insect-plant host associations. In this study we demonstrated that it is possible to detect and quantify with a high success rate specific PSMs associated with a particular plant host species. This method can be used as a means of confirming not only general insect phytophagy, but also in-situ plant host choice, including where multiple host plant species are available and have been fed upon. This innovative method overcomes the significant challenges associated with direct observation or low likelihood of finding plant DNA in sugar-only feeding insects. Given the importance of plant sugar feeding to a wide range of insect taxa, and the potentially complex but significant role of PSMs in fitness, survival and ecological interactions, knowledge of PSMs in nectar will potentially expand our understanding of the role of diet in the life cycles of insects, including those of medical importance.

## Methods and materials

### Plant selection and nectar chemistry analysis.

To determine PSM detectability in mosquitoes post-ingestion, known plant hosts were identified through a systematic review of studies exploring mosquito plant host selection, which identified 104 plant host species for mosquitoes (Appendix 1: Table S1)^21,22,26,28,38,45,53^. We identified three plants that were available for nectar collection: *Lantana camara* L., *Ricinus communis* L., and *Cascabela thevetia* (L.) Lippold (syn. *Thevetia peruviana*). Nectar sampling was carried out following the methods of Palmer-Young et al. (2019a). Samples were collected from *L*. *camara* and *R*. *communis* plants cultivated at the Royal Botanic Gardens, Kew. Nectar samples from *C*. *thevetia* were collected from populations adjacent to the Institut de Recherche en Sciences de La Santé (IRSS), Bobo Dioulasso, Burkina Faso with permission obtained. 5 µl nectar samples were collected with microcapillary tubes. A 95 µl aliquot of methanol was added to the nectar to prevent microbial growth to preserve chemical composition. Samples were analysed using Liquid Chromatography (LC)-Electrospray Ionization Mass Spectroscopy (ESIMS) with photodiode array using a Thermo Fisher Velos Pro LC‐MS. Samples (5 µl) were injected directly on to a Phenomenex Luna C18 (2) column (150 Å~3 mm i.d., 3 μm particle size at 400 µl min^− 1^ with column temperature at 30 °C and eluted using a linear gradient of 90:0:10 (t = 0 min) to 0:90:10 (t = 20–25 min), returning to 90:0:10 (t = 27–30 min) with water: methanol: 1% formic acid in acetonitrile, respectively. Compounds were detected on a Thermo Fisher Velos Pro Dual‐Pressure Linear Ion Trap Mass Spectrometer.

Three diagnostic PSMs were identified in the nectar: a pyridine alkaloid, ricinine, in *R*. *communis*; a flavone, luteolin, in *L*. *camara*; and a phenylpropanoid, thevefolic acid B, in *C*. *thevetia*. Ricinine (CAS 524-40-3) was detected from a LC-ESIMS peak eluted after a retention time of 5.60 min with *m/z* = 165 [M + H]^+^ and 329 [2 M + H]^+^, consistent with a molecular formula of C_8_H_8_N_2_O_2_. A peak for luteolin (CAS 491-70-3) was detected with a retention time of 15.00 min and *m/z* = 285 [M + H]^−^, consistent with a molecular formula of C_15_H_10_O_6_. Thevefolic acid B (CAS 87315-09-1) was detected with a retention time of 6.50 min and *m/z* = 225 [M-H]^−^, consistent with a molecular formula of C_10_H_10_O_6_. A mean concentration of ricinine in nectar was determined to be approximately 250 µM (0.04 g l-1), which was consistent with previous studies ^59,88,90,92–94,]^. The mean concentration for luteolin was 35 µM (0.01 g l^− 1^), which was previously observed in nectar^57,58,60^. The mean concentration for thevefolic acid B was determined to be 440 µM (0.1 g l-1), which has previously been identified in *C*. *thevetia*^56,65^.

### Experiment 1: plant secondary metabolite detection from dosed sugar solution

Adult mosquitoes were setup using 30 cm x 30 cm x 30 cm culture cages, three for each PSM treatment and one control cage. 7–10-day old males and females of *An*. *coluzzii* and *Cx*. *quinquefasciatus* (Appendix 2: Sect. 2) were used, 30 per treatment cages and 10 in the control cage. Cages were maintained in a climate-controlled insectary (25 ± 2° C, 60 ± 2% RH, 12:12 h light: dark). 24-hours prior to exposure, sugar-feeders were replaced with isotonic water. PSMs were provided to treatment mosquitoes in 10% sucrose solutions. Ricinine and thevefolic acid B were isolated from plants (Appendix 2: Sect. 1, Table S2, Figure S1) and luteolin was available commercially. PSM were added to the sugar solutions in the pre-determined concentrations of ricinine (250 µM), luteolin (35 µM) or thevefolic acid B (440 µM) for feeding ad libitum. Control mosquitoes were provided with sucrose only. Feeding treatments were maintained for three days, with dosed sugar solutions replaced daily. Dead mosquitoes were removed throughout, ranging from 0 to 5 dead of *Cx*. *quinquefaciatus* and 0 to 10 dead of *An*. *coluzzii*. Live mosquitoes were euthanized by freezing at -18 °C for 24 h.

Dead mosquitoes were sluiced with deionized water to remove residue from the outside of the mosquito. Groupings of five mosquitoes of each gender and sex were placed in Eppendorf tubes with 100 µl of methanol and a clean steel ball bearing. Due to mosquito mortality, treatment groups had at least 4 samples with 6 samples achieved when all mosquitoes survived. Tubes were sealed with parafilm, and samples were mashed by ball bearing on a shaker for 24 h. After mashing, samples were centrifuged, and the methanol solution removed by pipette for chemical analysis using LC-ESIMS, as described above. Target PSMs were identified from LC-ESIMS peaks and peak area was calculated. The 10 control samples from each species and sex were similarly prepared in two samples of 5 mosquitoes for LC-ESIMS analysis.

### Experiment 2: Floral and extra-floral assay

To determine if PSM could be detected post-ingestion from floral nectar feeding, a series of flower feeding assays were conducted at the Institut de Recherche en Sciences de la Santé (Bobo Dioulasso, Burkina Faso). The flower assays were conducted using one, two, or three plant species to explore further whether PSM detection is possible when mosquitoes have access to multiple plant hosts.

Laboratory-reared *An*. coluzzii were obtained from outbred colonies established in 2012 that have been repeatedly replenished with wild-caught F1 females, the same strain as mosquitoes used in Experiment 1, collected from Bobo Dioulasso, Burkina Faso (11°23’14"N, 4°24’42"W) and identified to species by PCR^110^. Mosquitoes were held in 30 cm × 30 cm × 30 cm mesh-covered cages under insectary conditions (12:12 h light: dark, 27 ± 2 °C, 70 ± 5% RH). Flowers from each plant species were obtained from Bobo Dioulasso, Burkina Faso: *C*. *thevetia* from parklands, *R. communis* and *L. camara* from the wild. Branches containing ten flowers were provided single-plant assays. Five flowers of each species were used in two-plant assays. Branches with four flowers of each plant were used in the three-plant assay. Flowers were harvested no more than 1 h before use to ensure that nectar would be present when provided to mosquitoes. The base of the branch was wrapped in moistened paper towels and aluminium foil to ensure that exudes from the cuts were not available for feeding. Flowers were provided to mosquitoes in 15 cm × 15 cm × 15 cm mesh-covered cages for a 12-hour period. At the end of the exposure period, mosquitoes were euthanized by freezing and placed in groups of 5 in Eppendorf tubes containing silica gel for shipment to RBG Kew for analysis. Mosquitoes were analysed using the same preparation and LC-ESIMS analysis described in experiment 1.

### Experiment 3: PSMs residence time

To determine how long PSMs could be detected post-ingestion, mosquitoes were fed until replete and then allowed to rest for a set time (0, 4, 8, 12, 24, or 48 h). Sugar solutions containing the target PSMs were prepared as they were in experiment 1. Brilliant blue food dye was also added to each treatment solution to facilitate visual inspection of the feeding progress. Male and female *An*. *coluzzii* and *Cx*. *quinquefasciatus*, which were reared according to the method described in experiment 1, were used. In preparation for feeding assays, pupae were separated from the larval trays into groups of less than 100 individuals of mixed sexes with *ad libitum* access to distilled water. Following emergence (24–48 h in *An*. *coluzzii*, 48–72 h in *Cx*. *quinquefasciatus*), the mosquitoes were placed in individual sugar feeding containers and offered one of three treatments. A strict feeding regime was followed, in which the mosquitoes imbibed the offered sugar solution until their abdomen was fully distended, typically achieved less than 10 min after feeding had commenced. Directly after feeding on prescribed treatment, mosquitoes were retained for time points 4–48 h in ‘resting vials’, individual 2 mL screw top vials with the screw top replaced by nylon mesh to allow ventilation. Treated mosquitoes were then maintained under laboratory conditions without sustenance but distilled water at 24 h intervals. At the time points 0, 4, 8, 24, and 48 h after feeding; the mosquitoes were euthanised on ice. The head, wings, and legs were removed, and the remaining sample placed in a glass vial and stored at − 20 °C until sample preparation. Mosquitoes belonging to the 0 h sample group were placed on ice directly after feeding. Chemical analyses of the individual mosquito samples were carried out following the method from experiment 1.

### Data analysis

To calculate the concentration of PSMs present in nectar and the mosquito samples, LC-ESIMS response curves were run using a set of reference samples of reference concentrations 10, 50 100, and 500 µg. For each PSM, the peak areas were plotted against the concentrations of the metabolites with the intercept set to zero. Calibration curves were calculated based on a peak area: parts per million relationship. The reference constants produced were then used in the following formula to calculate the weight of the metabolite in the samples:

$$\:(\text{Ps/Cs})\:\times1\times10^{-4}\:\text{L}\:=\:\text{w}\text{e}\text{i}\text{g}\text{h}\text{t}\:\left(\text{n}\text{g}\right)$$

Where Ps is the peak area of the sample metabolite and Cs is the constant produced from the reference. Mean weight for each set of male and female mosquitoes under each compound treatment were calculated.

## Electronic supplementary material

Below is the link to the electronic supplementary material.

Supplementary Material 1

Supplementary Material 2

## Data Availability

Data used in this study is available from the corresponding author upon reasonable request.
