# Supplementary material for: Identifying mosquito plant hosts from ingested nectar secondary metabolites
Source: Sci Rep. 2025 Feb 22;15:6488. doi: 10.1038/s41598-025-88933-1 (PMC11846922; doi:10.1038/s41598-025-88933-1)
Supplement: Supplementary file 2 — Supplementary Material 2 [file 41598_2025_88933_MOESM2_ESM.docx]

Appendix 2

Section 1: Mosquito Cultures

## Secondary metabolite isolation

For experiments 1 and 3, enough of each compound to provide artificial food with naturally occurring concentrations was required. Luteolin was acquired commercially from Sigma (UK). Ricinine was isolated from seeds of *R*. *communis*, which have high concentrations of ricinine (Holfelder et al., 1998). The ricinine isolation process, established at RBG Kew, used 200 g of *R*. *communis* seeds, which were split open, ground using a mortar and pestle, and then boiled for fifteen minutes (to deactivate the toxin ricin) in 1 litre of distilled water. The extract was then vacuum filtered with a coarse filter paper, which separated the seed remains from the liquid. The filtrate was re-filtered with a finer grade filter paper and then extracted with chloroform (100 ml) in a separating funnel and shaken for one minute. This was left to separate for 1-2 h. The chloroform was then run off and another 100 ml of chloroform was added, shaken, and left to separate. This process was repeated two additional times. After the last portion of chloroform was run off, the remaining aqueous layer was centrifuged for at least 20 min at 2000 rpm for further separation of chloroform and the aqueous layer. The combined chloroform extracts were evaporated to dryness under vacuum on a Buchi Rotovap (Buchi UK Ltd Newmarket, UK) and recrystallised from distilled water. Crystals were collected by vacuum filtration and left to dry in a desiccator. The compound identity was confirmed by High Performance Liquid Chromatography-High Resolution Mass Spectrometry (HPLC-HRMS) (experimental (+) m/z = 165.0652, calculated for C_8_H_8_O_2_N_2_ = 165.0658; uv: λmax 255, 306 nm).

Thevefolic acid B used in Experiments 1 and 2 was isolated from frozen *C*. *thevetia* leaves from wild populations collected from around Bobo Dioulasso, Burkina Faso. Leaves (10 g) were freeze dried, ground and boiled for 30 min in 100 mL of deionised water. This was then vacuum filtered whilst still hot (Whatman No. 1) and concentrated (Rotovap) to ca. 0.5 mL. Separation into fractions was achieved by flash chromatography (Isolera One) using 0% to 50% methanol-water, linear gradient, over 10 mins. The fractions were collected between 5-7 mins and were concentrated to dryness. The yield of this process was 11 mg (0.11%).

The structures of both the ricinine and thevefolic acid B were confirmed using NMR (Nair et al., 1987; de Melo Cazal et al., 2009). Spectra were collected from compounds solubilised in D_2_O at 30 °C on a Bruker Avance 400 MHz instrument. Standard pulse sequences and parameters were used to obtain one-dimensional 1H, 13C spectra for both ricinine and thevolic acid and two-dimensional gradient-enhanced COSY, HSQC, and HMBC spectra for thevefolic acid with 1,4-dioxane internal standard. Ricinine was confirmed by comparison with published data (Sousa et al., 2013) and thevefolic acid B by comparison with predicted resonances for this compound (Gunasegaran and Nair, 1985; Nair et al., 1987). Full NMR assignments for thevefolic acid B have not previously been reported and are provided here (Table S2, Figure S1).

Table S2. Thevefolic acid B NMR Assignments in D_2_O with 1,4-dioxane-d8

| Atom | **C/ppm** | **H/ppm** | **COSY** | **HMBC (weak** = w**)** |
| --- | --- | --- | --- | --- |
| CH(OH)-**C**OOH | 177.83 |  |  | 3.02, 3.22, 4.59 |
| arom-**C**OOH | 172.89 |  |  | 7.51, 7.45, 7.30w |
| 3 | 154.97 |  |  | 3.22, 3.02, 7.30,7.45w |
| 5 | 132.43 | 7.30 d *J*=8.0 |  | 3.22, 3.02, 7.51 |
| 4 | 130.40 |  |  | 7.45, 7.51, 4.59w |
| 1 | 130.35 |  |  | 7.3 |
| 6 | 122.26 | 7.51 dd *J*=8.0, 1.6 | 7.45 | 7.30, 7.45 |
| 2 | 116.76 | 7.45 d *J*=1.6 | 7.51 | 7.51 |
| 8 | 70.54 | 4.59 dd *J*=5.2, 8.0 | 3.22; 3.02 | 3.02, 3.22 |
| 7 | 35.34 | 3.22 dd *J*=14, 5.2 | 4.59; 3.02 | 4.59, 7.30 |
|  |  | 3.02 dd *J*=14, 8.0 | 4.59; 3.22 | 4.59, 7.30 |


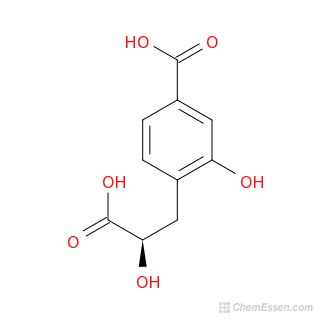

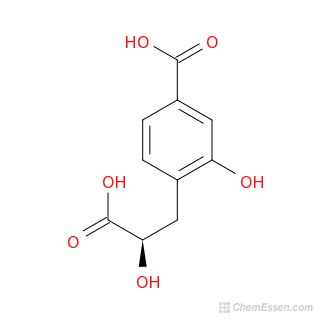


2

3

4

5

6

7

8

1

Figure S1. Structure of Thevefolic acid

Section 2: Mosquito Colonies used in Experiment 1 and 3

Both males and females of the malaria vector *An*. *coluzzii* (part of the *An*. *gambiae* sensu lato species complex) and the filarial nematode vector *Cx*. *quinquefasciatus* were used. Colonies of *An*. *coluzzii* were established at the Natural Resources Institute (NRI), UK, in 2006 from eggs derived from a colony reared in Burkina Faso and originally propagated from wild-caught blood-fed females. Colonies of *Cx*. *quinquefasciatus* were established at NRI from eggs derived from a long-term laboratory colony held at the London School of Hygiene and Tropical Medicine, UK. The colony has been in culture at NRI for over 10 years. All mosquitoes were reared in a climate-controlled insectary maintained at 26 ± 2 °C and 60 ± 10% RH, with a 12h:12h light:dark cycle. Adults were housed in 30 cm x 30 cm x 30 cm gauze cages and had *ad* *libitum* access to a solution of 10% sucrose dissolved in distilled water held in a wick feeder. Adult females were blood fed after 5-10 imaginal days: *An*. *coluzzii* were offered a blood meal from a human arm and oviposited on disks of filter paper dampened with 0.1% isotonic solution of aquarium salts and deionized water; *Cx*. *quinquefasciatus* were fed on defibrinated horse blood via a membrane feeding system (Hemotek, UK) and oviposited in dishes of isotonic water. Eggs were moved to trays of isotonic water for hatching, whereupon larvae of *An*. *coluzzii* were fed ground Tetramin Baby Fish Food (Tetrawerke, Melle, Germany), while larvae of *Cx*. *quinquefasciatus* were fed ground dog biscuits (Bakers® Weight Control, Purina, UK). Trays were inspected daily, and pupae separated for emergence in adult cages. Both colonies were free of human pathogens.

Section 3: Table **S3**. Average detected weight (in what ngs? Also, did this calculate include negative detections?) of each PSM over time in *An. coluzzii* and *Cx. quinquefasciatus,* ingested with a replete sucrose meal.

| ***An. coluzzii*** | | | | | | | |
| --- | --- | --- | --- | --- | --- | --- | --- |
|  |  | Female | | | Male | | |
|  | Time (h) | Mean | (SE) | # + | Mean | (SE) | # + |
| Luteolin | 0 | 3.51 | 0.85 | 5/5 | 4.53 | 1.15 | 5/5 |
|  | 4 | 2.30 | 0.85 | 5/5 | 1.90 | 0.77 | 5/5 |
|  | 8 | 0.41 | 0.18 | 3/5 | 0.70 | 0.18 | 5/5 |
|  | 24 | 0.06 | 0.02 | 4/5 | 0.02 | 0.02 | 1/3 |
|  | 48 | 0.04 | 0.04 | 1/2 | 0.03 | 0.03 | 1/2 |
|  | | | | | | | |
|  |  | Female | | | Male | | |
| Ricinine | Time (h) | Mean | (SE) | # + | Mean | (SE) | # + |
|  | 0 | 28.6 | 7.63 | 5/5 | 16.5 | 3.30 | 5/5 |
|  | 4 | 10.9 | 1.53 | 5/5 | 4.69 | 0.62 | 5/5 |
|  | 8 | 3.83 | 1.67 | 4/5 | 3.34 | 1.19 | 5/5 |
|  | 24 | 0 | 0 | 0/5 | 0 | 0 | 0/5 |
|  | 48 | 0 | 0 | 0/5 | 0 | 0 | 0/5 |
|  | | | | | | | |
|  |  | Female | | | Male | | |
|  | Time (h) | Mean | (SE) | # + | Mean | (SE) | # + |
| Thevefolic Acid B | 0 | 170 | 27.7 | 5/5 | 102 | 29.4 | 5/5 |
|  | 4 | 78.3 | 11.9 | 5/5 | 78.3 | 5.09 | 6/6 |
|  | 8 | 24.0 | 13.1 | 5/5 | 11.1 | 3.74 | 5/5 |
|  | 24 | 0 | 0 | 0/5 | 1.61 | 1.61 | 1/5 |
|  | 48 | 0.31 | 0.31 | 1/5 | 0 | 0 | 0/5 |
| ***Cx. quinquefasciatus*** | | | | | | | |
|  |  | Female | | | Male | | |
|  | Time (h) | Mean | (SE) | # + | Mean | (SE) | # + |
| Luteolin | 0 | 3.59 | 1.05 | 5/5 | 3.53 | 0.57 | 5/5 |
|  | 4 | 1.45 | 0.30 | 5/5 | 0.34 | 0.07 | 5/5 |
|  | 8 | 0.87 | 0.72 | 5/5 | 0.31 | 0.72 | 4/5 |
|  | 24 | 0.09 | 0.02 | 4/5 | 0.03 | 0.02 | 3/5 |
|  | 48 | 0.02 | 0.02 | 1/5 | 0.03 | 0.02 | 2/5 |
|  | | | | | | | |
|  |  | Female | | | Male | | |
|  | Time (h) | Mean | (SE) | # + | Mean | (SE) | # + |
| Ricinine | 0 | 23.5 | 3.80 | 5/5 | 19.6 | 1.76 | 5/5 |
|  | 4 | 11.3 | 4.82 | 5/5 | 11.3 | 3.77 | 5/5 |
|  | 8 | 8.43 | 3.47 | 5/5 | 0.88 | 0.39 | 5/5 |
|  | 24 | 0.27 | 0.16 | 3/5 | 0.14 | 0.04 | 4/5 |
|  | 48 | 0.08 | 0.05 | 2/5 | 0.25 | 0.12 | 4/5 |
|  | | | | | | | |
|  |  | Female | | | Male | | |
|  | Time (h) | Mean | (SE) | # + | Mean | (SE) | # + |
| Thevefolic Acid B | 0 | 280 | 43.1 | 5/5 | 181 | 17.4 | 5/5 |
|  | 4 | 126 | 42.7 | 5/5 | 136 | 46.6 | 5/5 |
|  | 8 | 76.4 | 23.2 | 5/5 | 25.9 | 7.26 | 5/5 |
|  | 24 | 6.35 | 6.35 | 1/6 | 0 | 0 | 0/5 |
|  | 48 | 0 | 0 | 0/2 | 0 | 0 | 0/2 |
